# Cold scissors versus electrosurgery for hysteroscopic adhesiolysis: A meta-analysis

**DOI:** 10.1097/MD.0000000000025676

**Published:** 2021-04-30

**Authors:** Liuqing Yang, Ling Wang, Yun Chen, Xiaoshi Guo, Chenyun Miao, Ying Zhao, Lu Li, Qin Zhang

**Affiliations:** a Department of TCM Gynecology, Hangzhou Hospital of Traditional Chinese Medicine Affiliated to Zhejiang Chinese Medical University; b The First Clinical College of Zhejiang Chinese Medical University; c College of Pharmaceutical Sciences, Zhejiang University, Xihu District, Hangzhou, Zhejiang, PR China.

**Keywords:** Asherman syndrome, electrosurgery, gynatresia, hysteroscopy, recurrence

## Abstract

**Background:**

Intrauterine adhesion seriously affects reproductive health in women. Hysteroscopic adhesiolysis using cold scissors or electrosurgery is the main treatment, although there is no consensus on the preferable method. This review aimed to compare the efficacy and safety of these methods for treating moderate to severe intrauterine adhesion.

**Methods:**

PubMed, EMBASE, MEDLINE, Cochrane Database of Systematic Reviews, Web of Science, Chinese Biomedical Literature Database, and China National Knowledge Infrastructure were searched on April 30, 2020. Randomized controlled trials and observational studies that were published in all languages (must contain English abstracts) and compared hysteroscopic cold scissors with electrosurgery for the treatment of intrauterine adhesion were included. Mean differences, odds ratios, and 95% confidence intervals (CIs) were reported. Bias was evaluated using the Cochrane Risk of Bias assessment tool for randomized controlled trials and the Newcastle-Ottawa Scale for observational studies. Data were analyzed using RevMan software (Review Manager version 5.3, The Cochrane Collaboration, 2014). Two researchers independently extracted data and assessed the quality of the included studies. If a consensus was not reached, a third researcher was consulted.

**Results:**

Nine studies (n = 761; 6 randomized controlled trials and 3 retrospective studies) were included. The intrauterine adhesion recurrence rate with second look hysteroscopy was significantly lower (odds ratio = 0.30, 95% CI = 0.16–0.56; *P* = .0002) with hysteroscopic cold scissors than with electrosurgery. The total operation time was significantly shorter (mean difference = –7.78, 95% confidence interval = –8.50 to –7.07; *P* < .00001), intraoperative blood loss was significantly lower (mean difference = –9.88, 95% CI = –11.25 to –8.51; *P* < .00001), and the menstrual flow rate was significantly higher (odds ratio = 4.36, 95% confidence interval = 2.56–7.43; *P* < .00001) with hysteroscopic cold scissors than with electrosurgery. There were no significant differences in the pregnancy rate. One complication (1 perforation case, hysteroscopic cold scissors group) was reported.

**Conclusions:**

Hysteroscopic cold scissors is more efficient in preventing intrauterine adhesion recurrence, increasing the menstrual flow, reducing intraoperative blood loss, and shortening the operation time.

## 1 Introduction

Intrauterine adhesion (IUA), or Asherman syndrome, occurs when the basal layer of the endometrium is damaged and replaced by fibrous tissue^[1]^; it clinically presents with hypomenorrhea, amenorrhea, and infertility. The primary cause of IUA is dilation and curettage after miscarriage.^[2]^ To date, the treatment of IUA remains challenging, although many postoperative adjuvant therapies are used to prevent adhesion.^[3,4]^

Hysteroscopy represents the gold standard method for diagnosing and treating IUA.^[5,6]^ Generally, there are 2 different methods used for hysteroscopic adhesiolysis (HA): electric and non-electric. Electric instruments have been demonstrated to be successful in the dissection of moderate and severe IUA.^[7]^ With the wide application of unipolar or bipolar instruments for HA, some specialists have realized that electrosurgery (ES) is like a double-edged sword. Although moderate and severe IUA is dissected with high efficiency, the thermal effect generated during the surgery will damage the remaining endometrium.^[8–11]^ Non-electric instruments mainly include cold scissors (CS),^[12]^ which do not release thermal energy and thus, avoid thermal injury to the endometrium.^[1,5,13]^ Nevertheless, some surgeons believe that scars are often too dense to cut with CS, and that it is difficult to achieve hemostasis.^[14]^

Compared with ES, hysteroscopy using mechanical tissue removal systems has major advantages for treating endometrial polyps and myomas.^[15–18]^ HA with CS has become increasingly common, with publication of a few randomized controlled trials (RCTs) on CS. Nevertheless, the efficacy and safety of CS have not been systematically evaluated. Thus, the objective of this meta-analysis was to compare CS and ES for the treatment of IUA with respect to the incidence of recurrent IUA, the operation time, intraoperative blood loss, the menstrual flow, the pregnancy rate, and complications.

## 2 Methods

### 2.1 Literature search

Relevant studies published before April 30, 2020 were systematically searched in PubMed, EMBASE, MEDLINE, Cochrane Database of Systematic Reviews, Web of Science, Chinese Biomedical Literature Database, China National Knowledge Infrastructure, and the Wangfang Data Knowledge Service Platform. The following search terms were used: “intrauterine adhesions,” “Asherman syndrome,” “intrauterine synechiae,” “hysteroscopy,” “hysteroscopies,” “hysteroscopic surgery,” “scissors,” “cold scissors,” “surgical scissors,” “cold knife,” and “micro scissors.” Supplemental Digital Content 1 shows the specific search strategy and screening process. The search was independently performed by three investigators (LY, LW, and YZ) without language or date restrictions. However, only articles with English abstracts were included for evaluation by the investigators. We also searched the references of the included studies to find additional studies for possible inclusion.

### 2.2 Eligibility criteria and study selection

The study inclusion criteria were as follows: study design, RCTs, or observational studies; patients, moderate to severe IUA confirmed by hysteroscopy; interventions, HA with CS (CS/cold knife/micro-scissors; CS or experimental group), or HA with ES (monopolar or bipolar electrosurgical system; control or ES group); and outcomes, at least one reported outcome (incidence of recurrent IUA with second look hysteroscopy, operation time, intraoperative blood loss, increased menstrual flow rate, pregnancy rate, and complications). The exclusion criteria were as follows: case reports, narrative reviews, correspondence articles, and articles without an English abstract. Duplicate articles were deleted using EndNote (X8). Titles and abstracts were independently screened by 2 investigators and irrelevant articles were discarded; the full texts of the remaining articles were acquired. When discrepancies occurred between the 2 investigators, a third investigator was consulted.

### 2.3 Data extraction

Two investigators extracted the following target data from the identified studies: lead author, year of publication, country, basic patient characteristics, study type and size of the studied cohort, and analysis outcomes, including the incidence of recurrent IUA at second look hysteroscopy, operation time, intraoperative blood loss, increased menstrual flow rate, pregnancy rate, and complications. Discrepancies between the 2 reviewers were resolved by discussion. If the data were not clear, the corresponding author was contacted by email for the missing information.

### 2.4 Quality assessment

Bias was evaluated by 2 investigators (LY, LW) using the Cochrane Risk of Bias assessment tool,^[19]^ which clarified the relative bias risk in each trial based on the following 6 judgment bias terms: selection, performance, detection, attrition, reporting, and others. The included studies were rated as having a low, high, or unclear risk of bias. Bias in the observational studies was evaluated by the Newcastle-Ottawa Scale (NOS),^[20]^ which is an ideal tool to assess the quality of cohort studies, including includes selection bias, comparability, and outcomes. The parameters under selection and outcome measures could be assigned 1 star at most, while comparability could be assigned 2 stars at most. In total, 9 stars could be awarded to an ideal study.

### 2.5 Ethical statements

No ethical approval is required since this is a literature-based study. The protocol of this meta-analysis has been registered with PROSPERO (no. CRD42020168008).

### 2.6 Statistical analyses

RevMan software (Review Manager version 5.3, The Cochrane Collaboration, 2014) was used to analyze the data extracted from the original articles. Binary variable outcomes (recurrent IUA, increased menstrual flow rate, and pregnancy rate) were analyzed using the odds ratio (OR), while continuous variables (operation time and intraoperative blood loss) were analyzed using the mean difference (MD). The result calculation used a 95% confidence interval (CI). Statistical heterogeneity among the studies was determined by Cochran *Q* test and the *I*^2^ index, in which *I*^2^ < 50% or *P* < .05 indicated the lack of significant heterogeneity. The fixed-effects model was used if there was no heterogeneity present among the studies; otherwise, the random-effects model was applied for pooled estimates. In addition, if there was high heterogeneity (*I*^2^ > 50%), we explored possible explanations for the heterogeneity through subgroup and sensitivity analyses. Potential publication bias was analyzed by plotting a funnel chart (see Figure, Supplemental Digital Content 2, which illustrates the symmetric distribution of the funnel plot).

## 3 Results

### 3.1 Study selection

**Figure 1:**
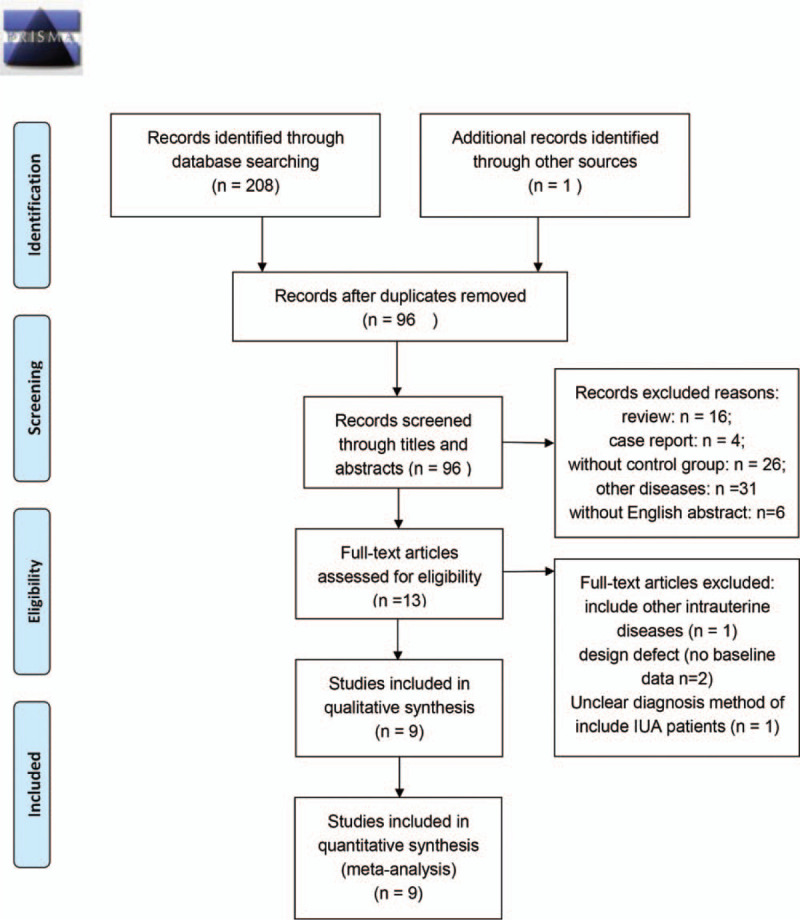
Preferred reporting items for systematic reviews and meta-analyses flow chart. IUA = intrauterine adhesions.

A total of 209 articles were identified according to the predefined search strategy (Fig. 1). From these, 113 duplicate articles were excluded. From the remaining 96 articles, 13 were included after reading of the titles and abstracts. These articles underwent full-text review, and 1^[21]^ was excluded because it included other intrauterine diseases, 2^[22,23]^ were excluded because the design was defective (without baseline data), and 1^[24]^ was excluded because of an unclear diagnosis method for IUA. Finally, 6 RCTs^[25–30]^ and 3 observational studies^[12,31,32]^ were included (Table 1).

**Table 1:** Results of the quality assessment using the Newcastle-Ottawa scale for retrospective studies.

	Selected					Exposure			
Study	Adequate definition of cases	Representativeness of the cases	Selection of controls	Definition of controls	Comparability control for the important factor	Ascertainment of exposure	Same method of ascertainment for cases and controls	Non-response rate	Score
Ai et al, 2017^[31]^	^∗^	^∗^	^∗^	^∗^	^∗∗^	^∗^	^∗^		8
He, 2014^[32]^	^∗∗^	^∗^	^∗^	^∗^	^∗∗^	^∗^	^∗^	^∗^	9
Zhao et al, 2019^[12]^	^∗^	^∗^	^∗^	^∗^	^∗∗^	^∗^	^∗^	^∗^	9

### 3.2 Main characteristics of the included articles

A detailed description of the characteristics of the 9 included studies, which involved 761 IUA patients, is presented in Table 2. Overall, 371 (48.8%) and 390 (51.2%) patients were included in the CS and ES groups, respectively. Five studies^[25,26,29–31]^ compared the incidence of recurrent IUA with second look hysteroscopy between CS and ES, 5^[25,26,28,29,32]^ reported the operation time, 2^[26,29]^ reported intraoperative blood loss, 6^[25–27,29,30,32]^ reported an increased menstrual flow rate in the third postoperative month, and 4^[12,27,29,32]^ reported postoperative pregnancy rates. Only one study^[27]^ reported complications. The follow-up periods in the studies ranged from 2 to 24 months.

**Table 2:**
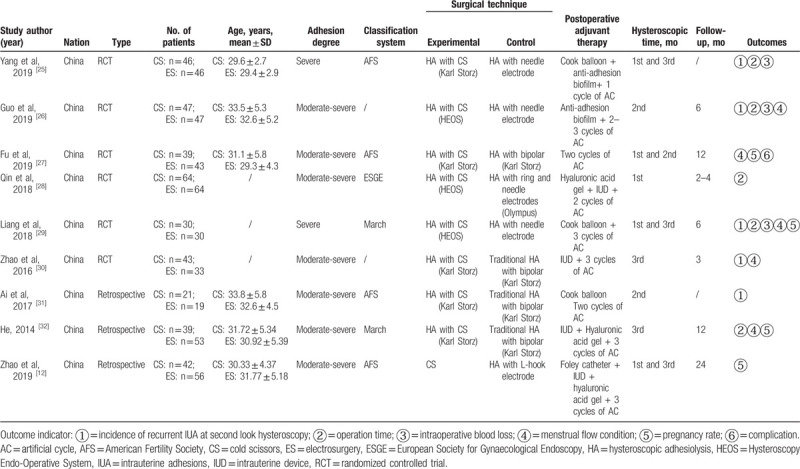
Main characteristics of the included studies.

### 3.3 Publication bias and evidence quality assessment

**Figure 2:**
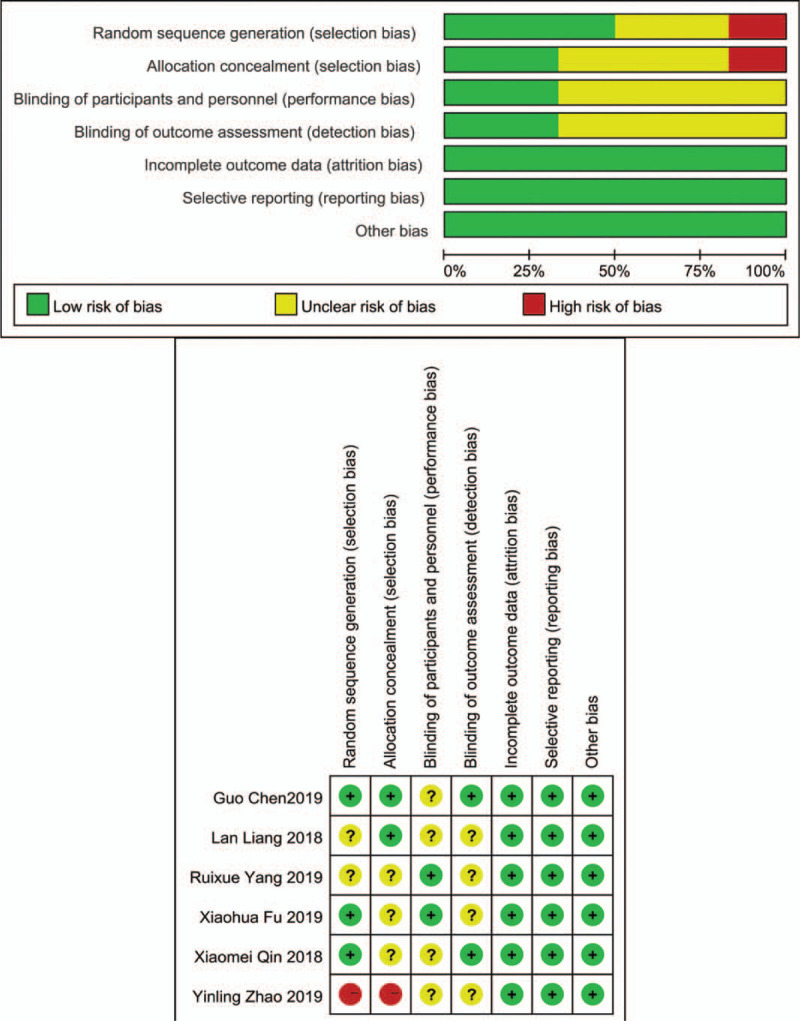
Bias of randomized controlled trials according to the Cochrane Risk of Bias assessment tool.

RCTs were evaluated using the Cochrane Risk of Bias assessment tool. The proportions of various biases in the included RCTs are shown in Fig. 2. A part of the bias domains was unclear in the 6 RCTs.^[25–30]^ According to the Cochrane quality assessment method, 4 RCTs had medium risk of bias and 2 had high risk of bias. The observational studies were assessed using the NOS and were found to have scores of 8 (selection: 4 stars, comparability: 2 stars, exposure: 2 stars), 9 (selection: 4 stars, comparability: 2 stars, exposure: 3 stars), and 9 (selection: 4 stars, comparability: 2 stars, exposure: 3 stars), respectively.

### 3.4 Results of the meta-analysis

#### 3.4.1 Incidence of recurrent IUA with second look hysteroscopy

**Figure 3:**
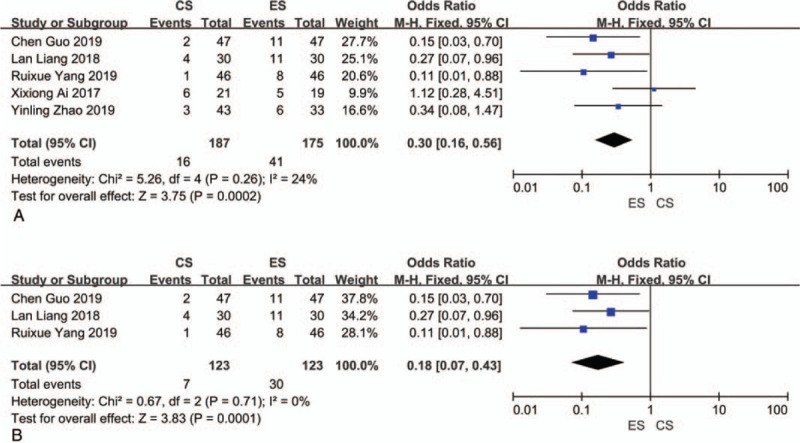
(A) Meta-analysis results for intrauterine adhesion (IUA) recurrence at second look hysteroscopy. (B) Subgroup analysis results for the incidence of IUA recurrence at second look hysteroscopy among severe grade IUA cases.

Five studies reported IUA recurrence with second look hysteroscopy,^[25,26,29–31]^ with a total sample size of 362. The results indicated low heterogeneity (*I*^2^ = 24%, *P* = .26). The results were combined using the fixed-effects model, and we found that the incidence of recurrent IUA was lower with CS than with ES (OR = 0.30, 95% CI = 0.16–0.56; *P* = .0002) (Fig. 3A). The results of subgroup analysis of the incidence of IUA recurrence at second look hysteroscopy among cases with severe IUA were consistent with the above results (OR = 0.18, 95% CI = 0.07–0.43; *P* = .0001) (Fig. 3B).

#### 3.4.2 Operation time

**Figure 4:**
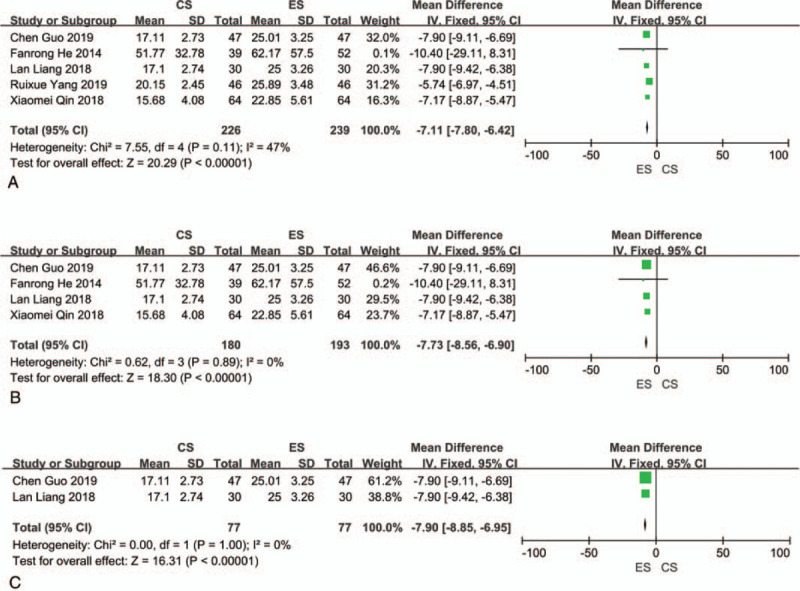
(A) Meta-analysis results for operation time. (B) Meta-analysis results for operation time after removing the article with a heterogeneous source. (C) Subgroup analysis results for operation time among severe grade IUA cases. IUA = intrauterine adhesions.

Five studies reported the operation time for both groups, with a total sample size of 465.^[25,26,28,29,32]^ The result showed moderately heterogeneity (*I*^2^ = 47%, *P* = .11) (Fig. 4A), probably due to the fact that all the doctors in the included studies, except 1,^[25]^ were from tertiary A hospitals (representative of large general hospitals in China). The gap between the surgical experience and technology may have affected the operation time, which, in turn, may have contributed to heterogeneity. After exclusion of that study,^[25]^ the heterogeneity disappeared. The operation time was shorter with CS than with ER (MD = –7.73, 95% CI = –8.56 to –6.90; *P* < .00001) (Fig. 4B). The results of subgroup analysis of the operation time among cases of severe IUA cases were consistent with the above results (MD = –7.90, 95% CI = –8.85 to –6.95; *P* < .00001) (Fig. 4C).

#### 3.4.3 Intraoperative blood loss

**Figure 5:**
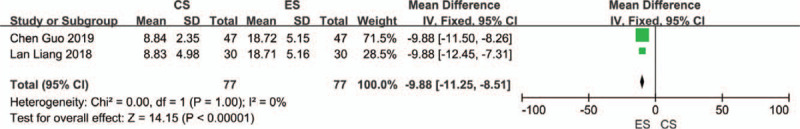
Meta-analysis results for intraoperative blood loss.

Two studies reported intraoperative blood loss for both procedures, with a total sample size of 154.^[26,29]^ The result showed no significant heterogeneity (*I*^2^ = 0%, *P* = 1.00). The results were combined using the fixed-effects model. Intraoperative blood loss was lower in the CS group than in the ES group (MD = –9.88, 95% CI = –11.25 to –8.51; *P* < .00001) (Fig. 5).

#### 3.4.4 Increased menstrual flow rate

**Figure 6:**
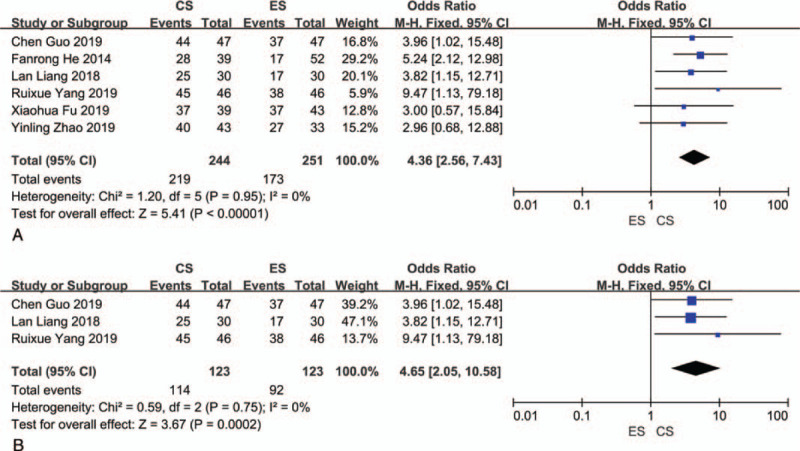
(A) Meta-analysis results for the increased menstrual flow rate. (B) Subgroup analysis results for menstrual flow at the third postoperative month among severe grade IUA cases. IUA = intrauterine adhesions.

Six studies compared the menstrual flow at the third postoperative month, with a total sample size of 495 and no heterogeneity in the results (*I*^2^ = 0%, *P* = .95).^[25–27,29,30,32]^ The results were combined using the fixed-effects model. The increased menstrual flow rate was significantly lower in the ES group than in the CS group (OR = 4.36, 95% CI = 2.56–7.43; *P* < .00001) (Fig. 6A). The results of subgroup analysis for cases of severe IUA were consistent with the above results (OR = 4.65, 95% CI = 2.05–10.58; *P* = .0002) (Fig. 6B).

#### 3.4.5 Pregnancy rate

**Figure 7:**
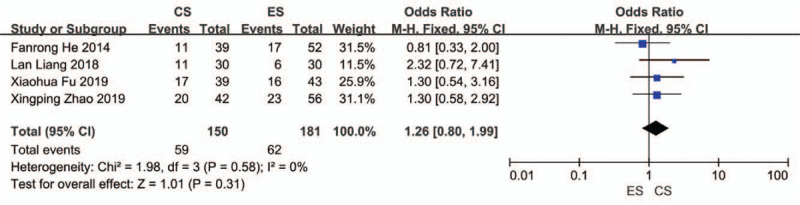
Meta-analysis results for pregnancy rate.

Four studies reported the postoperative pregnancy rates for both groups, with a total sample size of 331.^[12,27,29,32]^ The results indicated no significant heterogeneity (*I*^2^ = 0%, *P* = .58) and were combined using the fixed-effects model. There was no statistically significant difference in postoperative pregnancy rates (OR = 1.26, 95% CI = 0.80–1.99; *P* = .31) (Fig. 7).

#### 3.4.6 Complications

Only one complication was found in this review; specifically, there was 1 case of perforation case in the CS group.^[27]^ With regard to complications, the meta-analysis failed because of limited data.

#### 3.4.7 Subgroup and sensitivity analyses

Because of the insignificant heterogeneity, subgroup analysis was not used to investigate the sources of heterogeneity. The results were closely related to adhesion grades.^[7]^ Therefore, subgroup analysis of severe IUA was performed (Figs. 3B, 4C, 6B). However, the included studies did not separately list the postoperative results for moderate IUA; this precluded subgroup analysis of moderate IUA. Sensitivity analysis was conducted to investigate the dependability of the results by excluding studies one by one. After the exclusion of each study, the results did not change. Thus, this meta-analysis was less sensitive, and the results were credible.

## 4 Discussion

A major long-term complication that all surgeons attempt to avoid during hysteroscopic surgery is postoperative IUA, which can impair reproductive outcomes. In general, the IUA recurrence rate was found to be lower with CS than with ES. Mazzon et al^[33]^ reached the same conclusion and reported that IUA was less frequent with cold loop hysteroscopic myomectomy than with ES. One possible reason for the higher IUA recurrence rate with ES is the injury to the endometrium caused by the energy-based instrument.^[8–11]^ The specific mechanism underlying endometrial injury due to ES may involve local hypoxia, reduced neovascularization, and increased expression of inflammatory cytokines and fibroblast growth factors in the endometrium.^[34–36]^

This study's pooled meta-analysis showed that, compared with ES, CS was associated with a shorter procedure duration. CS can avoid bubble formation and provide a clear visual field for the surgeon, and this can reduce the operation time. Although the decrease in the total procedure duration might not be clinically significant, it could increase patient acceptance and reduce intraoperative complications. The intraoperative blood loss estimated by the surgeon could be somewhat subjective. However, the blood loss in both groups was low and completely within the safe range.

We also found that, compared with ES, CS significantly improved the menstrual flow rate. Menstruation recovery is determined by endometrial function and increased menstrual flow after surgery, and it can be a potential predictor of restored endometrial function.^[37]^ We found that the increased menstrual flow rate was lower in the ES group than in the CS group, probably because the heat energy produced by the electric instruments may have caused some damage to the endometrium.

Endometrial angiogenesis is essential for good endometrial receptivity for embryo implantation. The amount of menstrual flow may also reflect the growth status of endometrial blood vessels and function.^[38]^ Zhao et al^[4]^ and Yu et al^[11]^ consistently found that the improved menstrual flow after hysteroscopic surgery had a significant positive relationship with the pregnancy rate. Our meta-analysis showed that CS could improve the menstrual flow rate and reduce IUA recurrence. Therefore, we speculate that CS is beneficial for embryo implantation and can increase the pregnancy rate. Although there was no significant difference in the pregnancy rate between the CS and ES groups, we believe that this result was influenced by the small number of studies. Thus, further high-quality RCTs are needed to further evaluate the impact of the 2 surgical methods on the pregnancy rate after HA.

We found 1 case of perforation in the CS group. A previous study reported that uterine perforation was one of the most common complications of operative hysteroscopy.^[39]^ However, damage by electric instruments may lead to more serious injuries.^[40]^ When uterine perforation occurs during the use of electrosurgical electrodes, it is necessary to identify bowel abnormalities using laparoscopy. Some experts have suggested that the use of mechanical instruments to treat uterine diseases (polyps, adhesions, and myomas, among others) may help in preventing visceral injury.^[41]^

## 5 Strengths and limitations

This review provided the first comprehensive analysis of the effectiveness of electric and non-electric instruments in the treatment of IUA. The potential limitations of this meta-analysis should be considered. First, there were differences in postoperative adjuvant therapies (artificial cycle, intrauterine balloons, and intrauterine devices, among others) between the 2 groups. Because of the limitations in the existing literature, we could only overlook these differences and explore the curative effect of different instruments (CS and ES) for IUA treatment. Second, other details (operators’ techniques) might have also led to bias. Third, considering our objectives and restrictive criteria, we were unable to conduct extensive analyses, including evaluations of cost-effectiveness, patient tolerance, and pregnancy outcomes. More RCTs of relatively high quality need to be conducted in the future.

## 6 Conclusions

Based on available evidence, this meta-analysis showed that CS was more effective than ES in preventing IUA recurrence, increasing the menstrual flow, reducing intraoperative blood loss, and shortening the operation time. Further high-quality trials are required to assess the efficacy and safety of CS and ES.

## Acknowledgments

The authors would like to thank Daisy Chow for English language editing.

## Author contributions

**Conceptualization:** Liuqing Yang.

**Data curation:** Liuqing Yang, Ling Wang, Ying Zhao.

**Formal analysis:** Liuqing Yang, Ling Wang, Yun Chen, Lu Li.

**Funding acquisition:** Qin Zhang.

**Investigation:** Yun Chen.

**Supervision:** Qin Zhang.

**Writing – original draft:** Liuqing Yang, Ling Wang, Xiaoshi Guo, Chenyun Miao, Ying Zhao.

**Writing – review & editing:** Liuqing Yang, Ling Wang, Xiaoshi Guo, Chenyun Miao, Ying Zhao, Lu Li, Qin Zhang.

## Supplementary Material

Supplemental Digital Content

## Supplementary Material

Supplemental Digital Content
