# Supplementary material for: Cold scissors versus electrosurgery for hysteroscopic adhesiolysis: A meta-analysis
Source: Medicine (Baltimore). 2021 Apr 30;100(17):e25676. doi: 10.1097/MD.0000000000025676 (PMC8084071; doi:10.1097/MD.0000000000025676)
Supplement: Supplemental Digital Content [file medi-100-e25676-s001.pdf]

Supplemental Digital Content 1. The specific search strategy and screening process

1) PubMed

Search Results: 25 Items

Search strategy: ((((((((((Gynatresias) OR (Asherman Syndrome)) OR (Syndrome, Asherman)) OR (Intrauterine Synechiae)) OR (Synechiae, Intrauterine)) OR (Uterine Synechiae)) OR (Synechiae, Uterine)) OR (Asherman's Syndrome)) OR (Ashermans Syndrome)) OR (Syndrome, Asherman's)) AND (((((((((((((((Hysteroscopy) OR (Hysteroscopies)) OR (Uterine Endoscopy)) OR (Uteroscopy)) OR (Uteroscopies)) OR (Endoscopy, Uterine)) OR (Endoscopies, Uterine)) OR (Uterine Endoscopies)) OR (Hysteroscopic Surgical Procedures)) OR (Hysteroscopic Surgical Procedure)) OR (Procedure, Hysteroscopic Surgical)) OR (Procedures, Hysteroscopic Surgical)) OR (Surgical Procedure, Hysteroscopic)) OR (Surgery, Hysteroscopic)) OR (Surgical Procedures, Hysteroscopic)) OR (Hysteroscopic Surgery))) AND (((((((((((((scissors) OR (cold scissors)) OR (surgical scissors)) OR (cold knife)) OR (micro-scissors)) OR (surgical instrument)) OR (forceps)) OR (Scissors, Surgical)) OR (Surgical Hooks)) OR (Hooks, Surgical)) OR (bipolar)) OR (monopolar)) OR (unipolar)) OR (electrosurgical resection))

2) Embase

Search Results: 10 Items

No.	Query	Results
#44	#7 AND #18 AND #42 AND #43	10
#43	#36 OR #37 OR #38 OR #39 OR #40 OR #41	130589
#42	#19 OR #20 OR #21 OR #22 OR #23 OR #24 OR #25 OR #26 OR #27 OR #28 OR #29 OR #30 OR #31 OR #32 OR #33 OR #34 OR #35	43889
#41	'electrosurgical resection'	51
#40	unipolar	14712
#39	monopolar	7718
#38	bipolar	118236
#37	'gastrointestinal biopsy device'/exp	1
#36	'endoscopic monopolar electrosurgical electrode'/exp	0
#35	'micro shear'	112
#34	'micro scissors'	31

#33	'cold knife'	1057
#32	'forceps'	24144
#31	hook	16534
#30	'surgical hooks'	5
#29	'surgical hook'	295
#28	'surgical scissors'	73
#27	'surgical plug'	2
#26	'surgical plugs'	0
#25	'surgical clips'	805
#24	'surgical clip'	353
#23	'surgical clamps'	16
#22	'surgical clamp'	46
#21	'scissor forcep'	2
#20	'scissor forceps'	23
#19	'scissors'/exp	1778
#18	#8 OR #9 OR #10 OR #11 OR #12 OR #13 OR #14 OR #15 OR #16 OR #17	11957
#17	'hysteroscopic surgeries'	24
#16	'hysteroscopic surgery'	604
#15	'hysteroscopic surgical procedure'	1
#14	'hysteroscopic surgical procedures'	16
#13	'uterine endoscopies'	0
#12	uteroscopies	0
#11	uteroscopy	18
#10	'uterine endoscopy'	6
#9	hysteroscopies	522
#8	'hysteroscopy'/exp	11790
#7	#1 OR #2 OR #3 OR #4 OR #5 OR #6	1919

#6	'ashermans syndrome'	9
#5	'intrauterine synechiae'	102
#4	gynatresias	4
#3	'asherman syndrome'	201
#2	'intrauterine adhesions'	752
#1	'uterus synechia'/exp	1468

3) MEDLINE:

Search Results: 83 Items

NO.	QUERY	Result
S4	S1 AND S2 AND S3	(47)
S3	scissors OR cold scissors OR surgical scissors OR cold knife OR micro scissors OR surgical instrument OR surgical hooks OR forceps OR bipolar OR monopolar OR unipolar OR electrosurgical resection	(34,5086)
S2	hysteroscopy OR Hysteroscopic OR uterine endoscopy OR uteroscopy OR uterine endoscopies OR hysteroscopic surgical procedures OR hysteroscopic surgical procedure OR hysteroscopic surgery OR hysteroscopic surgeries	(13,120)
S1	intrauterine adhesions OR ashermans syndrome OR gynatresias OR intrauterine synechiae OR ashermans syndrome	(1,482)

4) The Cochrane Central Register of Controlled Trials (CENTRAL):

Search Results: 7 Items

ID	Search	Hits
#1	MeSH descriptor: [Gynatresia] explode all trees	18
#2	(Syndrome, Asherman):ti,ab,kw OR (Synechiae, Intrauterine):ti,ab,kw OR (Intrauterine Synechiae):ti,ab,kw OR (Syndrome, Asherman's):ti,ab,kw OR	88

	(Gynatresias):ti,ab,kw (Word variations have been searched)	
#3	MeSH descriptor: [Hysteroscopy] explode all trees	395
#4	(Uteroscopies):ti,ab,kw OR (Uterine Endoscopy):ti,ab,kw OR (Uteroscopy):ti,ab,kw OR (Hysteroscopies):ti,ab,kw OR (Endoscopies):ti,ab,kw (Word variations have been searched)	12732
#5	(Endoscopy, Uterine):ti,ab,kw OR (Uterine Endoscopies):ti,ab,kw OR (Surgical Procedures):ti,ab,kw OR (Hysteroscopic):ti,ab,kw OR (Procedure, Hysteroscopic Surgical):ti,ab,kw (Word variations have been searched)	41069
#6	(Hysteroscopic Surgeries):ti,ab,kw OR (Surgeries, Hysteroscopic):ti,ab,kw OR (Hysteroscopic Surgical Procedures):ti,ab,kw OR (Hysteroscopic Surgical Procedure):ti,ab,kw OR (Surgery, Hysteroscopic):ti,ab,kw (Word variations have been searched)	454
#7	(Procedures, Hysteroscopic Surgical):ti,ab,kw OR (Hysteroscopic Surgery):ti,ab,kw OR (Surgical Procedure, Hysteroscopic):ti,ab,kw (Word variations have been searched)	454
#8	MeSH descriptor: [Surgical Instruments] explode all trees	729
#9	(Surgical Scissors):ti,ab,kw OR (Scissors):ti,ab,kw OR (bipolar):ti,ab,kw OR (monopolar):ti,ab,kw OR (unipolar):ti,ab,kw (Word variations have been searched)	9712
#10	(electrosurgical resection):ti,ab,kw (Word variations have been searched)	74
#11	#1 OR #2	88
#12	#3 OR #4 OR #5 OR #6 OR #7	52365
#13	#8 OR #9 OR #10	10436
#14	#11 AND #12 AND #13	7

5) Web of Science

Search Results: 7 Items

Intrauterine Adhesions AND surgical scissors

6) China Knowledge Resource Integrated (CNKI)

Search Results: 56 Items

We submitted Chinese words for “Intrauterine Adhesions” AND “hysteroscopy” AND “scissors OR micro scissors OR cold scissors OR cold knife” in all fields

7) Chinese Biomedical Literature Database:

Search Results: 58 Items

We submitted Chinese words for “Intrauterine Adhesions” AND “hysteroscopy” AND “scissors OR micro scissors OR cold scissors OR cold knife” in all fields

Total papers from databases: 23+10+47+7+7+56+58=208 items

8) Hand Searching

One paper was included from hand searching by other reviews.

9) Literature Screening

All studies were imported to EndNote X8. After discarding the duplicates, 96 studies were remained. After title and abstract screening 83 papers were excluded and 13 papers were remained. After full-text screening 4 studies were excluded. The remaining 9 studies were included in the meta-analysis.

The 13 studies included or excluded in the full-text screening are listed as follows:

NO.	Full-text articles assessed for eligibility	Inclusion/ exclusion	Reason for exclusion
1	Huang Y, Xie M. Comparison of the effect of hysteroscopic cold knife and traditional bipolar electrosurgical knife in the separation of intrauterine adhesions. Fujian Med J. 2019;41:85-87.	EX	incomplete data
2	Wang J, He S, Chen Q, Che Y. Efficacy of different surgical methods under hysteroscope in treating intrauterine adhesions. The Chinese Journal of Human Sexuality. 2019;28:78-80.	EX	incomplete data
3	Zhang M. The short-term effect of microsurgical scissors separation in the treatment of recurrent intrauterine adhesions. HENAN JOURNAL OF SURGERY. 2019;25:143-144.	EX	incomplete data
4	Zhou J, Chen J, Weng T, Cheng F, Zhu K, Pan Q. Application and advantage of hysteroscopic cold instruments. China Journal of Endoscopy.2019;25;103-107.	EX	include other intrauterine diseases
5	Yang R, Tang L, Liu X. Effect of hysteroscopic	IN	N/A

	STORZ microscissor separation on the therapeutic effect of patients with severe Intrauterine Adhesions. Life Science Instruments. 2019;17:96-100.		
6	Guo C, Wang L, Wang L, Cao H. Clinical study on resection using micro-scissors under HEOS for intrauterine adhesion. Journal of Minimally Invasive Medicine. 2019;14:48-50.	IN	N/A
7	Fu X, Ji M, Shu J. The implement of micro-scissor for hysteroscopic adhesiolysis in reproductive medicine center. Journal of Wenzhou Medical University. 2019;49:52-55.	IN	N/A
8	Zhao Y, Qian H, Chen M, Xu G. Clinical effect comparison of two kinds of hysteroscopy in the treatment of moderate and severe intrauterine adhesion. Chinese Community Doctors. 2016;32:46-47.	IN	N/A
9	Qin X, Li L, Qin G, Jiang X. Comparative study on the effect of intrauterine adhesion cold knife surgery and electric cautery on endometrium. Chin J Reprod Contracep. 2018;38:481-484.	IN	N/A
10	Ai X, Li M, Zeng H, Zeng Z, Liang X. Reproductive Outcome Analysis of Two Kinds of Hysteroscopic Adhesiolysis. J Int Obstet Gynecol. 2017;44:594-598.	IN	N/A
11	Zhao X, Zhang A, Gao B, Arvind B, Huang H, Xu D. Cold scissors ploughing technique in hysteroscopic adhesiolysis:a comparative study. Ann Transl Med. 2019.	IN	N/A
12	Liang L, Zhao R, Hu X, Y F. The effect of HEOS hysteroscopic sharp separation in the treatment of sever uterine adheesion. Chin J Laparoscopic Surgery (Electronic Edition). 2018;11:103-106.	IN	N/A
13	He F. Retrospective comparative study of two surgical treatments of patiets with moderate to severe intrauterine adhesions. Guangzhou: Gynecology of	IN	N/A

Chinese Medicine, Guangzhou University of Chinese Medicine; 2014.
