# Supplementary material for: Cold scissors versus electrosurgery for hysteroscopic adhesiolysis: A meta-analysis
Source: Medicine (Baltimore). 2021 Apr 30;100(17):e25676. doi: 10.1097/MD.0000000000025676 (PMC8084071; doi:10.1097/MD.0000000000025676)

Supplemental Digital Content 2. Figure that illustrates the symmetric distribution of the funnel plot.


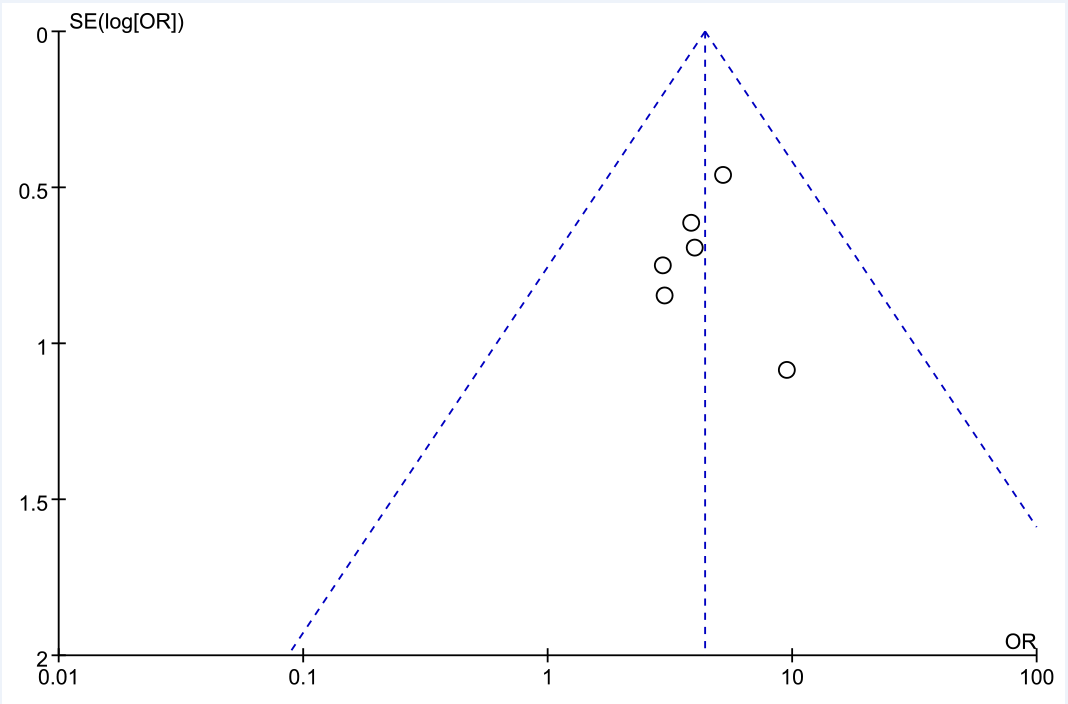

Supplement: Supplemental Digital Content [file medi-100-e25676-s002.doc]
